# Kv2 dysfunction after peripheral axotomy enhances sensory neuron responsiveness to sustained input^☆^

**DOI:** 10.1016/j.expneurol.2013.11.011

**Published:** 2014-01

**Authors:** Christoforos Tsantoulas, Lan Zhu, Ping Yip, John Grist, Gregory J. Michael, Stephen B. McMahon

**Affiliations:** a Neurorestoration Group, Wolfson Centre for Age-Related Diseases, King's College London, London SE1 1UL, UK; b Centre for Neuroscience & Trauma, Blizard Institute, Bart's and The London School of Medicine and Dentistry, Queen Mary University of London, London E1 2AT, UK

**Keywords:** AP, action potential, APD50, AP half width, AHPD50, after-hyperpolarization half width, ATF3, activating transcription factor 3, CGRP, calcitonin gene-related peptide, CNS, central nervous system, DRG, dorsal root ganglion, GAPDH, glyceraldehyde 3-phosphate dehydrogenase, IB4, isolectin B4, IHC, immunohistochemistry, IR, input resistance, ISH, *in situ* hybridization, Kv channel, voltage-gated potassium channel, NF200, neurofilament 200, RP, refractory period, ScTx, stromatoxin-1, SNT, spinal nerve transection, Neuropathic pain, Potassium channel, Dorsal root ganglia

## Abstract

Peripheral nerve injuries caused by trauma are associated with increased sensory neuron excitability and debilitating chronic pain symptoms. Axotomy-induced alterations in the function of ion channels are thought to largely underlie the pathophysiology of these phenotypes. Here, we characterise the mRNA distribution of Kv2 family members in rat dorsal root ganglia (DRG) and describe a link between Kv2 function and modulation of sensory neuron excitability. Kv2.1 and Kv2.2 were amply expressed in cells of all sizes, being particularly abundant in medium-large neurons also immunoreactive for neurofilament-200. Peripheral axotomy led to a rapid, robust and long-lasting transcriptional Kv2 downregulation in the DRG, correlated with the onset of mechanical and thermal hypersensitivity. The consequences of Kv2 loss-of-function were subsequently investigated in myelinated neurons using intracellular recordings on *ex vivo* DRG preparations. In naïve neurons, pharmacological Kv2.1/Kv2.2 inhibition by stromatoxin-1 (ScTx) resulted in shortening of action potential (AP) after-hyperpolarization (AHP). In contrast, ScTx application on axotomized neurons did not alter AHP duration, consistent with the injury-induced Kv2 downregulation. In accordance with a shortened AHP, ScTx treatment also reduced the refractory period and improved AP conduction to the cell soma during high frequency stimulation. These results suggest that Kv2 downregulation following traumatic nerve lesion facilitates greater fidelity of repetitive firing during prolonged input and thus normal Kv2 function is postulated to limit neuronal excitability. In summary, we have profiled Kv2 expression in sensory neurons and provide evidence for the contribution of Kv2 dysfunction in the generation of hyperexcitable phenotypes encountered in chronic pain states.

## Highlights

• Kv2.1 and Kv2.2 are expressed in rat dorsal root ganglion neurons.
• Kv2 subunits are most abundant in myelinated sensory neurons.
• Kv2.1 and Kv.2 subunits are downregulated in a traumatic nerve injury pain model.
• Kv2 inhibition *ex vivo* allows higher firing rates during sustained stimulation.
• We conclude that Kv2 channels contribute to limiting peripheral neuron excitability.

## Introduction

Chronic neuropathic pain is associated with profound changes in the anatomy and function of sensory neurons. One of the most extensively documented, but not well understood, consequences of direct nerve injury in animal models and human subjects is the subsequent increase of sensory neuron excitability, primarily manifested as spontaneous discharge and increased responsiveness to stimulation (Kajander and Bennett, 1992, Liu et al., 1999, Study and Kral, 1996, Zhang et al., 1997). This injury-mediated hyperexcitability is thought to underlie poorly managed chronic symptoms in patients, such as spontaneous pain and hypersensitivity to stimulation.

Voltage-gated potassium (Kv) channels play a vital role in neuronal function by regulating resting membrane potential and controlling the waveform and frequency of APs (Hille et al., 1999). Indeed, injury-induced Kv dysfunction is linked to reduction of associated currents, augmented sensory neuron excitability and pain phenotypes (Chien et al., 2007, Everill and Kocsis, 1999, Tan et al., 2006, Tsantoulas et al., 2012). Accordingly, Kv blocker application to the DRG induces neuronal firing (Kajander et al., 1992), while Kv openers restrict neuronal excitability and relieve pain symptoms (Blackburn-Munro and Jensen, 2003, Dost et al., 2004, Mishra et al., 2012, Roza and Lopez-Garcia, 2008).

In many neurons, delayed rectifying currents due to Kv2 conductance (Guan et al., 2007, Malin and Nerbonne, 2002, Murakoshi and Trimmer, 1999) are a key modulator of excitability by facilitating AP repolarisation and inter-spike hyperpolarisation during repetitive firing (Blaine and Ribera, 2001, Johnston et al., 2010, Malin and Nerbonne, 2002). The Kv2 family consists of the Kv2.1 and Kv2.2 subunits (Frech et al., 1989, Hwang et al., 1992, Swanson et al., 1990). In the central nervous system (CNS) Kv2.1 features activity-dependent localisation and function (Misonou et al., 2004, O'Connell et al., 2010) and has a paramount role in regulating somatodendritic excitability, especially during high frequency input (Du et al., 2000, Misonou et al., 2005). Additional Kv2.1 functional diversity is achieved through interaction with modulatory Kv subunits (Bocksteins et al., 2012, Hugnot et al., 1996, Kerschensteiner and Stocker, 1999, Kramer et al., 1998, Sano et al., 2002, Stocker et al., 1999, Vega-Saenz de Miera, 2004) and auxiliary proteins (Leung et al., 2003, Peltola et al., 2011), while some studies have also proposed non-conducting roles (Deutsch et al., 2012, Feinshreiber et al., 2010, O'Connell et al., 2010, Pal et al., 2003, Redman et al., 2007). Although there is substantially less knowledge on Kv2.2, the high degree of conservation between the two subunits suggests common characteristics. Indeed, Kv2.2 mediates membrane hyperpolarization during trains of APs (Johnston et al., 2008, Malin and Nerbonne, 2002) and can associate *in vitro* with modulatory Kv subunits in a similar fashion to Kv2.1 (Fink et al., 1996, Hugnot et al., 1996, Salinas et al., 1997a, Salinas et al., 1997b).

Despite the recognised prominent role of Kv2 channels in shaping CNS excitability, no expressional or functional profiling in the periphery has been performed yet. As a result, the Kv2 involvement in sensory neuron excitability and in pain processing in particular remains unknown. Here, we characterized the Kv2 distribution in the DRG and examined the effect of nerve injury on Kv2 expression and function. In addition, we investigated whether pharmacological Kv2 modulation can recapitulate excitability changes linked to chronic pain states.

## Methods

### Animals and surgery

Adult male Wistar rats (200–250 g, Harlan Labs) were used in all experiments. All animal procedures conformed to institutional guidelines and the United Kingdom Home Office Animals (Scientific Procedures) Act 1986. Experimental neuropathy was induced by L5 spinal nerve transection (SNT, n = 8), using the method previously described (Kim and Chung, 1992). Briefly, a small incision on the skin overlaying left side L5–S1 was made and the vertebral transverse processes were exposed after retraction of the paravertebral musculature. The L6 transverse process was partially removed using bone rongeurs and the L5 spinal nerve was identified, tightly ligated and sectioned 1–2 mm distal to the ligature. The wound was cleaned with saline and the overlying muscles and skin were sutured. For dorsal rhizotomy (n = 3), a hemi-laminectomy was performed at the cervical level and the central processes of three consecutive DRGs (C5–C7) were identified and cut with fine iridectomy scissors. The wound was cleaned with saline and sutured at both muscle and skin levels. Animals were allowed to recover in a temperature-regulated chamber before returned to the home cage.

### Behavioural studies

Behavioural experiments were performed by a single experimenter, blinded to the identity of surgery the animals received. All tests were conducted in a quiet, temperature controlled room (22 °C). Animals were allowed to acclimatize for 15 min or until exploratory behaviour ceased before testing commenced. Mechanical allodynia was assessed using a von-Frey filament connected to a Dynamic Plantar Aesthesiometer (Ugo Basile). Each rat was placed in a ventilated plexiglass cage (22 × 16.5 × 14 cm) upon an elevated aluminium screen surface with 1 cm mesh openings. An actuator filament (0.5 mm diameter) under computer control delivered a linear stimulation ramp of 2.5 g/s to the plantar surface of the hind paw. Withdrawal thresholds were averaged over three consecutive tests with at least 5 min intervals in between measurements. A cut-off of 50 g was imposed to avoid the risk of tissue damage. Thermal response latencies were determined using the method previously described (Hargreaves et al., 1988). Briefly, each animal was placed into a clear ventilated plexiglass cage (22 × 16.5 × 14 cm) with a glass floor. A thermal challenge from a calibrated (190 mW/cm^2^) radiant light source was applied to the hindpaw until a withdrawal reflex was recorded. Withdrawal latencies were averaged over three consecutive tests, at least 5 min apart from each other. A cut-off of 20 s was imposed to prevent the possibility for tissue damage.

### Tissue preparation for histology

When tissue was destined for *in situ* hybridization (ISH), all preparation steps were carried out using ribonuclease (RNAse)-free or diethylpyrocarbonate (DEPC, Sigma)-treated reagents and equipment to minimize mRNA degradation. Rats were transcardially perfused under pentobarbitone anaesthesia with heparinized saline followed by fixation with freshly made 4% paraformaldehyde in 0.1 M phosphate buffer, pH 7.4. DRGs were removed and post-fixed in the perfusion fixative for 2 h. Tissue was then equilibrated in 20% sucrose in 0.1 M phosphate buffer (pH 7.4) at 4 °C overnight, embedded in O.C.T. compound. Tissue was cut at 8 μm thickness on a cryostat, and sections were thaw-mounted onto Superfrost Plus glass slides (VWR).

### Immunohistochemistry

When combined with *in situ* hybridization, immunohistochemistry (IHC) was performed first using RNAse-free or DEPC-treated materials and antibody solutions were supplemented with 100 U/ml RNasin Plus ribonuclease inhibitor (Promega). For IHC, sections were incubated overnight at RT with the appropriate primary antibody solution in PBS supplemented with 0.2% Triton X-100 and 0.1% NaN_3_ (PBS-Tx-Az). Primary antibodies used in this study were mouse anti-β3tubulin (1:2000, Promega), rabbit anti-ATF3 (1:200, Santa Cruz Biotechnologies), rabbit anti-CGRP (1:2000, Sigma), mouse anti-NF200 (1:500, Sigma) and rabbit anti-glial fibrillary acidic protein (rabbit anti-GFAP, 1:1000, DakoCytomation). Secondary antibodies were added for 4 h and were donkey anti-mouse AlexaFluor 488 and donkey anti-rabbit AlexaFluor 546 (1:1000, Invitrogen). IB4 detection was performed by using biotin-conjugated IB4 (1:200, Sigma) and AMCA Avidin D (1:400, Vector Labs).

### *In situ* hybridization

ISH was performed using 34-nucleotide long probes, as previously described in detail (Michael et al., 1997). Probe sequences were Kv2.1: tctggtttcttcgtggagagtcccaggagttcca, and Kv2.2: catccaaaggtctatccccacgagttcccaagca, complementary to bases 1954–1987 and 2650–2683 of kcnb1 (NM_013186.1) and kcnb2 (NM_054000.2) mRNAs, respectively. Probes were radioactively end-labelled with ^35^S-dATP (Perkin-Elmer) and unincorporated nucleotides were removed with a Sephadex G50 DNA chromatography column (GE Healthcare). Following pre-hybridization treatments (acetylation in 0.1 M triethanolamine/0.025 M acetic anhydride, dehydration in graded alcohols, chloroform dilipidation, ethanol rehydration), probe was added on sections overnight at 37 °C. The hybridization buffer composition was 2 × Denhardt's solution (Sigma), 20 × standard saline citrate, 50% deionised formamide, 10% dextran sulphate (Pharmacia Biotech), 100 μg/ml poly A (Sigma), 100 μg/ml sheared salmon sperm DNA (Boehringer), 20 μg/ml tRNA (Sigma) and 20 mM DTT. The following day, slides were washed in salt solutions with increasing stringencies to remove unspecific labelling (2 × 15 min in 2 × SSC/β-ME at RT, 2 × 15 min in 1 × SSC at 50 °C, 1 × 15 min in 0.2 × SSC at 50 °C, 2 × 20 min in 1 × SSC at RT, 0.1 × SSC), dehydrated and air-dried. Slides were dipped in autoradiographic emulsion (LM1, GE Healthcare), stored away at 4 °C in sealed boxes with silica gel and developed after 3–4 weeks using developer (Kodak D19, 2.5 min), stop (0.5% acetic acid, Sigma) and fix (25–40% sodium thiosulphate, 2 × 5 min, BDH) solutions. Unless combined with IHC, slides were counterstained with 0.1% Toluidine blue (Sigma) and coverslipped with DPX mounting medium (BDH). As a control, a 100-fold excess of unlabelled oligonucleotide was added to the hybridization reaction, which effectively competed all specific binding of radiolabeled probe. Further confidence in the specificity of detection was drawn by comparing distribution patterns using separate probes to the mRNAs of interest. Sequences for these additional control probes were Kv2.1: gtgtcaagttgaagaaagccgagcaggactggag, and Kv2.2: ctatgttttgctcaggcgtatggctcccatgcag.

### Image analysis

Visualisation and image acquisition were performed on a Leica fluorescence microscope fitted with polarized light block for epi-fluorescence. Analysis of signal intensity for each cell was carried out with ImageJ software to determine cell positivity for mRNA expression. Briefly, an area of interest (ROI) was drawn around the cell using the outline tool, and the mean silver grain density within this ROI was calculated. A neuron was considered positive when its mean silver grain density was greater than image background density (averaged from 3 separate ROIs over slide background) plus two times the standard deviation of this density. All quantitative measurements were done using a 25 × objective from at least five ganglion sections per animal (300–700 cell profiles, n = 3–4). Cell diameter (D) was indirectly calculated from whole ROI area (Α) using the formula D = 2√(A / π), while digital pixels were converted into μm units using a calibrated microscopic image taken at the same magnification. Measurements of cell diameter were only carried out on cells featuring a clearly visible nucleus, to ensure the section plane was near the middle of the cell and thus measurement would be representative of cell size. Assessment of Kv co-localisation with neuronal markers was performed by taking counts at 25 × magnification from at least five DRG sections per animal (300–1500 cell profiles, n = 3–4).

### qRT-PCR

Rats were sacrificed and L4–L5 DRGs from control or injured animals were rapidly (< 10 min) removed and snap-frozen in liquid nitrogen. Samples were homogenized in RLT buffer (Qiagen) using a table-top homogenizer and total RNA was isolated using an RNeasy Mini Kit (Qiagen). During RNA extraction residual genomic DNA was removed by RNase-free DNase treatment (Qiagen). First strand cDNA was reverse-transcribed from 1 μg of total RNA, using Superscript II Reverse Transcriptase, reaction buffer, DTT (all from Invitrogen), random primers and dNTP mix (Promega), according to the manufacturer's guidelines. Quantitative PCR was performed using the standard curves method (eight 3-fold dilution series of E15/E16 rat brain cDNA). All samples were run in triplicates and glyceraldehyde 3-phosphate dehydrogenase (GAPDH) was used as internal control to compensate for reverse transcription and amplification efficiency variation. Sequences for primers used were: Kv2.1: (F)-cggagaagaaccacttcgag, (R)-ttcatgcagaactcagtggc; Kv2.2: (F)-gctgcagttccagaatgtga, (R)-aatgatggggataggaaggg; and GAPDH: (F)-atggccttccgtgttcctac, (R)-agacaacctggtcctcagtg (all written 5′–3′). Primers were designed with Primer3 software and submitted to BLAST analysis to ensure annealing specificity. For template amplification, 20 ng cDNA/reaction was subjected to the following cycling conditions: (i) 95 °C for 10 min, (ii) denaturation at 95 °C for 15 s, annealing and extension at 60 °C for 60 s (40 cycles) and (iii) melting-curve temperature ramp to 105 °C. Amplification signal was detected using SYBR Green 1 dye (Roche) on a Rotor-Gene 3000 thermal cycler and transcript levels were quantified with Rotor-Gene 6 software (Corbett Life Science). Control reactions with water produced no amplification signal and melting curve analysis confirmed specificity of the products.

### Intracellular recordings

Naive (n = 18) and injured (5–9 days post SNT surgery, n = 6) animals were used for this experiment. On the day of recording, the animal was anaesthetised with an i.p. injection of urethane (25% w/v, 1.5 g/Kg, Sigma) and L4/L5 DRGs connected to the dorsal root and spinal nerve were dissected and transferred to a recovering chamber. The chamber was filled with constantly oxygenated calcium-free Kreb's solution containing (in mM) 124 NaCl, 26 NaHCO_3_, 1.3 NaH_2_PO_4_, 2 MgCl_2_, 2 CaCl_2_, 3 KCl, and 10 glucose. An hour after recovery, the tissue was incubated in 0.125% (w/v) collagenase (Sigma) in F12 medium (Invitrogen) at 37 °C for 20 min and then transferred to a recording chamber constantly oxygenated with 2 mM CaCl_2_ containing Kreb's solution as above. The ganglion was immobilised with U-shaped pins and the end of dorsal root was subjected to stimulation with a suction electrode. Recordings from DRG neurons were made with a sharp electrode pulled from filamented borosilicate glass (OD 1.5 mm × ID 0.86 mm, Sutter instrument). The pipette resistance was 25–40 MΩ when filled with 3 mM KCl. An axoclamp 2B amplifier (Molecular Devices) was used, analogue signals were low-pass filtered at 3 kHz and sampled at 5 kHz using a Power 1401 computer interface and data was acquired using Signal software (CED). Following cell impaling, a dorsal root stimulation evoked AP was obtained. To measure the refractory period, a paired-pulse (200 μs wide, 2 × dorsal root threshold current) stimulation was delivered to the dorsal root with a gradually shortened interval (coarse step of 1 ms and final step of 0.1 ms) until the second AP failed. In the experiment examining AP conduction probability, a train of 80 stimuli (200 μs wide, 2 × dorsal root threshold current) was delivered to the dorsal root at frequencies of 100, 200, 250, and 333 Hz in the absence and presence of ScTx. Recordings where stimuli trains induced AP conduction failure were included in the analysis. The conduction probability was calculated as a ratio of the number of evoked APs to the number of stimuli delivered. An averaged ratio from various frequency trains represents the AP conduction probability for that cell. ScTx (100 nM, Alomone Labs) was applied for at least 4 min before protocols commenced as normal. A small negative pulse (− 0.5 nA, 20 ms) was used to monitor input resistance (IR) throughout the experiment and sessions in which IR fluctuated more than 20% or resting membrane potential depolarized to more than − 45 mV were discarded from analysis. Data was analysed using Signal (CED) and Clampfit (Molecular Devices). Values represent mean ± SEM and paired *t*-tests were used for statistical analysis.

## Results

### Kv2.1 and Kv2.2 mRNA expression in sensory neurons

**Fig. 1:**
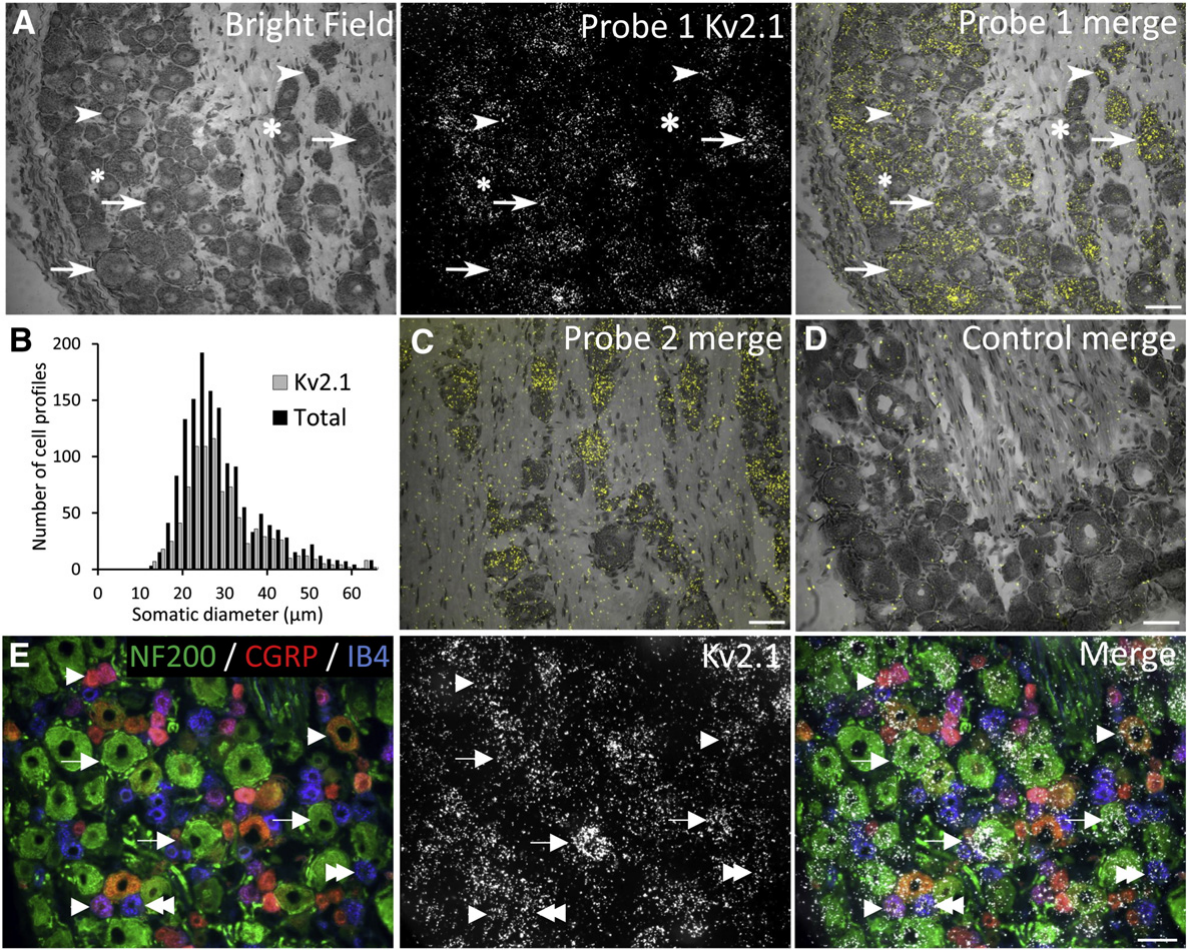
Kv2.1 mRNA expression in rat DRG neurons. (A) Bright field and ISH signal for Kv2.1 in naïve lumbar DRG from rat. Merged image illustrates Kv2.1 mRNA expression in a mixture of medium-large (arrows) and small (arrowheads) neurons. Asterisks denote examples of cells negative for Kv2.1 mRNA. (B) Kv2.1 cell-size distribution in lumbar DRG neurons. (C) Using a second probe for Kv2.1 gave identical detection patterns. (D) Control hybridizations show only background signal and confirm the specificity of the reaction. (E) NF200 (green), CGRP (red) and IB4 (blue) immunoreactivity (left) and Kv2.1 ISH (middle) in naïve lumbar DRG sections. Overlaid image demonstrates Kv2.1-positive neurons also co-labelling for NF200 (arrows), CGRP (arrowheads) or IB4 (double arrowheads). Scale bars = 50 μm.

**Fig. 2:**
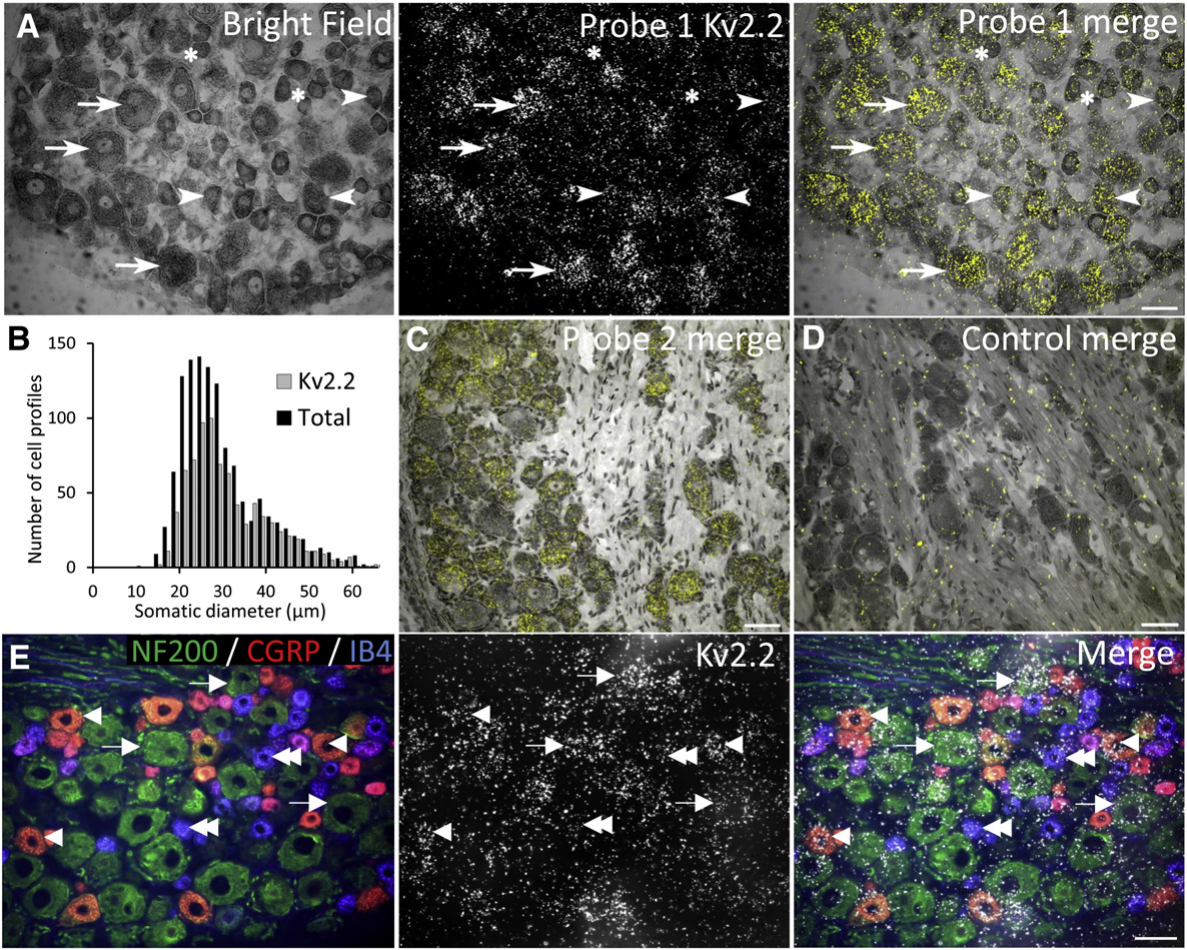
Kv2.2 mRNA expression in rat DRG neurons. (A) Bright field and polarized light images of Kv2.2 silver grains in rat lumbar DRG. Overlaid image demonstrates Kv2.2 expression in the vast majority of medium-large diameter neurons (arrows), as well as many small diameter (arrowheads) cells. Asterisks denote examples of cells negative for Kv2.2 mRNA. (B) Kv2.2 cell-size distribution in naïve DRG neurons. (C) Similar signal distribution using a second Kv2.2 mRNA probe. (D) Competition of labelling control reaction. (E) DRG section stained with antibodies against neuronal markers (left) and Kv2.2 mRNA signal (middle). Overlay identifies Kv2.2-positive neurons that are immunoreactive for NF200 (arrows), CGRP (arrowheads) or IB4 (double arrowheads). Scale bars = 50 μm.

We initially examined Kv2 subunit expression in naïve lumbar DRG using *in situ* hybridization. Approximately 62.7% and 61.3% of all DRG neurons expressed Kv2.1 and Kv2.2 mRNAs, respectively. Kv2.1 could be detected in a mixture of cells (Fig. 1A), being more abundant in medium (76.9%) and large (72.2%) neurons (arrows) but also present in more than half of small neurons (55.4%, arrowheads). Of all Kv2.1-positive neurons, 45.1% were medium-large and 54.9% were small in diameter. Kv2.2 ISH (Fig. 2A) revealed a similar distribution pattern in small neurons (45.0%, arrowheads) but this mRNA was even more highly expressed in medium (90.0%) and large (92.2%) neurons (arrows). In the total Kv2.2-positive population, 53.0% were medium-large and 47.0% small size. The above findings are reflected in the Kv2.1 (Fig. 1B) and Kv2.2 (Fig. 2B) cell-size distribution graphs, while a quantitative summary of the respective counts is presented in Table 1, Table 2. Hybridizations with a second probe targeted against separate mRNA regions of Kv2.1 or Kv2.2 mRNA gave similar patterns of expression (Figs. 1C & 2C, respectively), while negative control reactions involving competition with a non-labelled probe produced only background levels (Figs. 1D & 2D).

**Table 1:** Summary of Kv2.1 mRNA cell-size distribution counts.

Cell size	DRG neurons ± SE (%)	
	Kv2.1(+) cells in each class	Allocation of Kv2.1(+) cells within each class
Small (< 30 μm)	55.4 ± 3.2	54.9 ± 3.3
Medium (30–40 μm)	76.9 ± 1.8	31.0 ± 1.6
Large (> 40 μm)	72.2 ± 0.3	14.1 ± 3.9

**Table 2:** Summary of Kv2.2 mRNA cell-size distribution counts.

Cell size	DRG neurons ± SE (%)	
	Kv2.2(+) cells in each class	Allocation of Kv2.2(+) cells within each class
Small (< 30 μm)	45.0 ± 3.3	47.0 ± 2.8
Medium (30–40 μm)	90.0 ± 3.5	34.1 ± 3.2
Large (> 40 μm)	92.2 ± 1.3	18.9 ± 5.2

We next examined co-localisation of Kv2 subunits with known markers of neuronal subpopulations in the DRG, namely calcitonin gene-related peptide and isolectin B4 (CGRP and IB4, indicating peptidergic and non-peptidergic nociceptors respectively) and neurofilament 200 (NF200, expressed by myelinated neurons). By combining immunohistochemistry for these markers with ISH for Kv2.1 (Fig. 1E) and Kv2.2 (Fig. 2E) we could localise Kv2 mRNA in NF200-positive (arrows) and CGRP-immunoreactive (arrowheads) or IB4-binding (double arrowheads) neurons. Kv2.1 signal was found in 80.4%, 42.9% and 34.5% of neurons labelling for NF200, CGRP or IB4, respectively. In the Kv2.1-positive population, the majority of cells were immunoreactive for NF200 (60%), while a smaller proportion stained for CGRP (25.2%) or IB4 (19.1%) (Table 3). Performing a similar analysis, Kv2.2 signal was detected in 71.4%, 42.7% and 48.7% of NF200, CGRP or IB4-positive neurons, respectively. Of all cells labelled for Kv2.2 mRNA, 64.2% were also positive for NF200, 20.1% for CGRP, and 27.6% for IB4 (Table 4).

**Table 3:** Counts of Kv2.1 mRNA co-localisation with DRG neuronal subgroups.

Marker	DRG neurons ± SE (%)	
	Kv2.1(+) cells in each group	Allocation of Kv2.1(+) cells within each group
CGRP	49.2 ± 0.5	25.2 ± 2.5
IB4	34.5 ± 2.6	19.1 ± 1.2
NF200	80.4 ± 1.3	60.0 ± 1.2

**Table 4:** Counts of Kv2.2 mRNA distribution in DRG neuronal subpopulations.

Marker	DRG neurons ± SE (%)	
	Kv2.2(+) cells in each group	Allocation of Kv2.2(+) cells within each group
CGRP	42.7 ± 2.6	20.1 ± 3.6
IB4	48.7 ± 5.6	27.6 ± 2.4
NF200	71.4 ± 1.4	64.2 ± 2.4

In summary, the histological assessment illustrates that the Kv2.1 and Kv.2 subunits are widely expressed in a mixture of DRG neurons and appear enriched in the myelinated neuron population.

### Regulation of Kv2 subunits by nerve lesions

**Fig. 3:**
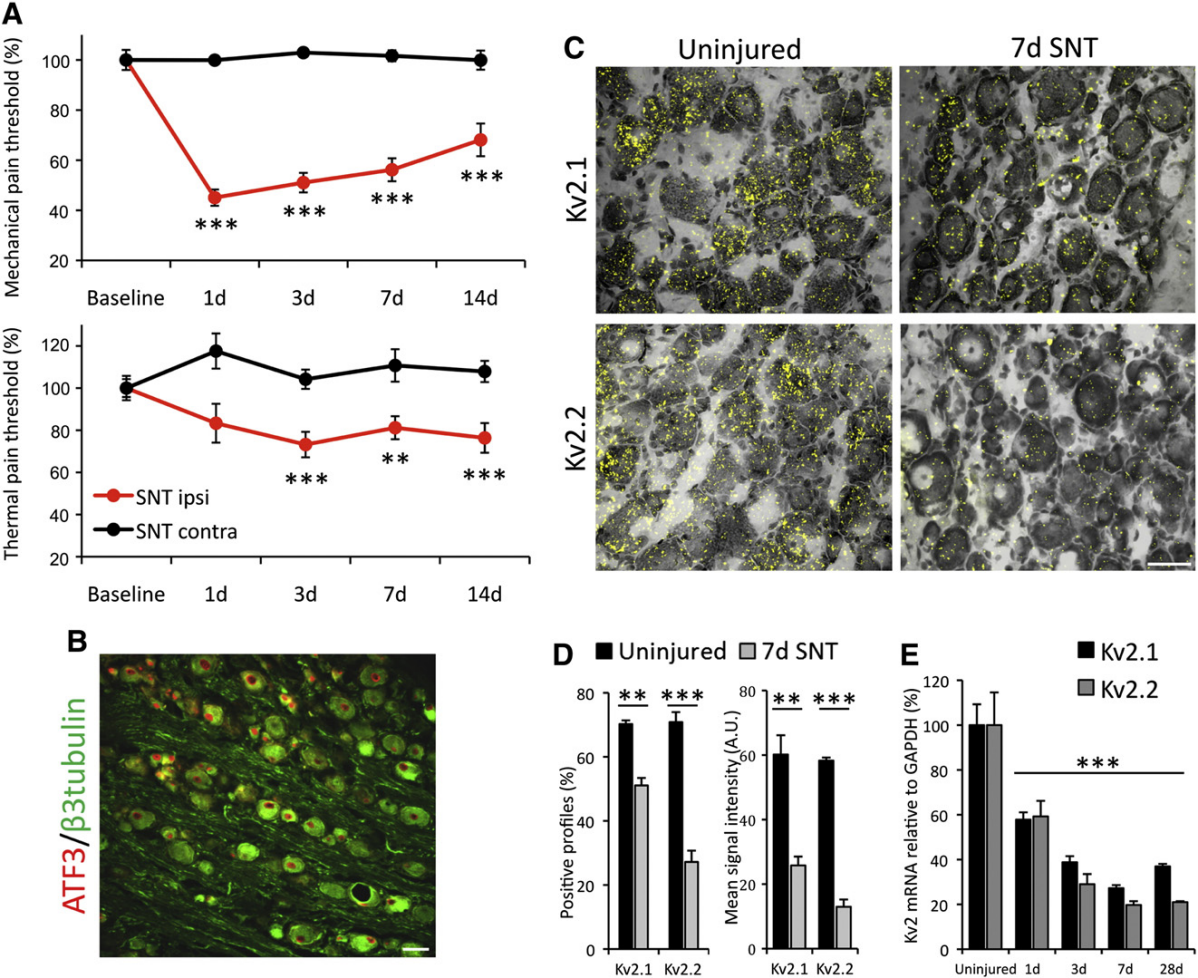
Kv2 subunits are reduced by peripheral axotomy. (A) Development of mechanical allodynia (top) and thermal hyperalgesia (bottom) on the ipsilateral hindpaw of SNT animals, but not on the contralateral side (mean ± SEM, n = 8, two-way ANOVA with Tukey's, **p < 0.01, ***p < 0.001 *vs* baseline). (B) Lumbar DRG stained for β3tubulin and ATF3, 7 days after SNT surgery. Virtually all L5 DRG neurons feature an injured phenotype, evident by upregulation of ATF3 expression in the nucleus. (C) Overlaid images of Kv2.1 (top) and Kv2.2 (bottom) mRNA hybridization in DRG neurons from uninjured (left) or SNT (right) animals, 7 days after axotomy. (D) Percentage neurons expressing Kv2 mRNA (left) and quantification of ISH signal intensity (right) in control and SNT animals, 7 days after axotomy (mean ± SEM, n = 3 animals/group, unequal variance *t*-test for each subunit, ***p < 0.001, **p < 0.01). (E) qRT-PCR quantification of Kv2 downregulation time-course after peripheral injury (mean ± SEM, n = 3, one-way ANOVA with Tukey's, ***p < 0.001 compared to uninjured for each subunit). Scale bars = 50 μm.

Having established the Kv2 expression profile in naïve sensory neurons, we then sought to examine regulation of Kv2 subunits by peripheral injury. For this, we used axotomy introduced by L5 spinal nerve transection (SNT), a well-established animal model of chronic pain. Following the insult animals developed robust and long-lasting mechanical allodynia on the injured (SNT ipsi), but not on the spared (SNT contra) side, as assessed by von-Frey testing (Fig. 3A, top). In addition, animals exhibited thermal hyperalgesia with a similar time-course (Fig. 3A, bottom). Following SNT surgery, virtually all L5 neurons showed strong nuclear immunostaining for the nerve injury marker ATF3 (Fig. 3B), confirming successful and complete axotomy of these neurons.

We then investigated the effect of axotomy on Kv2 mRNA expression, at a time where pain behaviours are established in the SNT model (Fig. 3C). When compared to sham controls (left panels), the ISH signal for Kv2.1 and Kv2.2 was substantially decreased at 7 d post-axotomy (right panels), both in terms of percentage (Kv2.1, 27.2% reduction; Kv2.2, 61.7% reduction; p < 0.01, n = 3, unequal variance *t*-test; Fig. 3D, left) and signal intensity (Kv2.1, 57.2% reduction; Kv2.2, 77.8% reduction; p < 0.01, n = 3, unequal variance *t*-test; Fig. 3D, right). In order to analyse the time-course of this downregulation in more detail, we quantified Kv2 mRNA levels by qRT-PCR (Fig. 3E), which revealed significant transcriptional downregulation of both Kv2.1 and Kv2.2 by axotomy. More specifically, mRNA levels for both subunits were significantly reduced by approximately 50% at 24 h after injury and continued to decrease reaching minimum levels at 7 d (Kv2.1, 73 ± 1.3% reduction; Kv2.2, 80 ± 1.7% reduction; p < 0.001 compared to uninjured for each subunit, n = 3, one-way ANOVA with Tukey's,). Thus, the emergence of pain phenotypes in the SNT model was correlated with decreases in Kv2 mRNA expression. Of note, some residual expression could be detected after 28 d, which could be exploited for compensatory treatments with Kv2 openers.

**Fig. 4:**
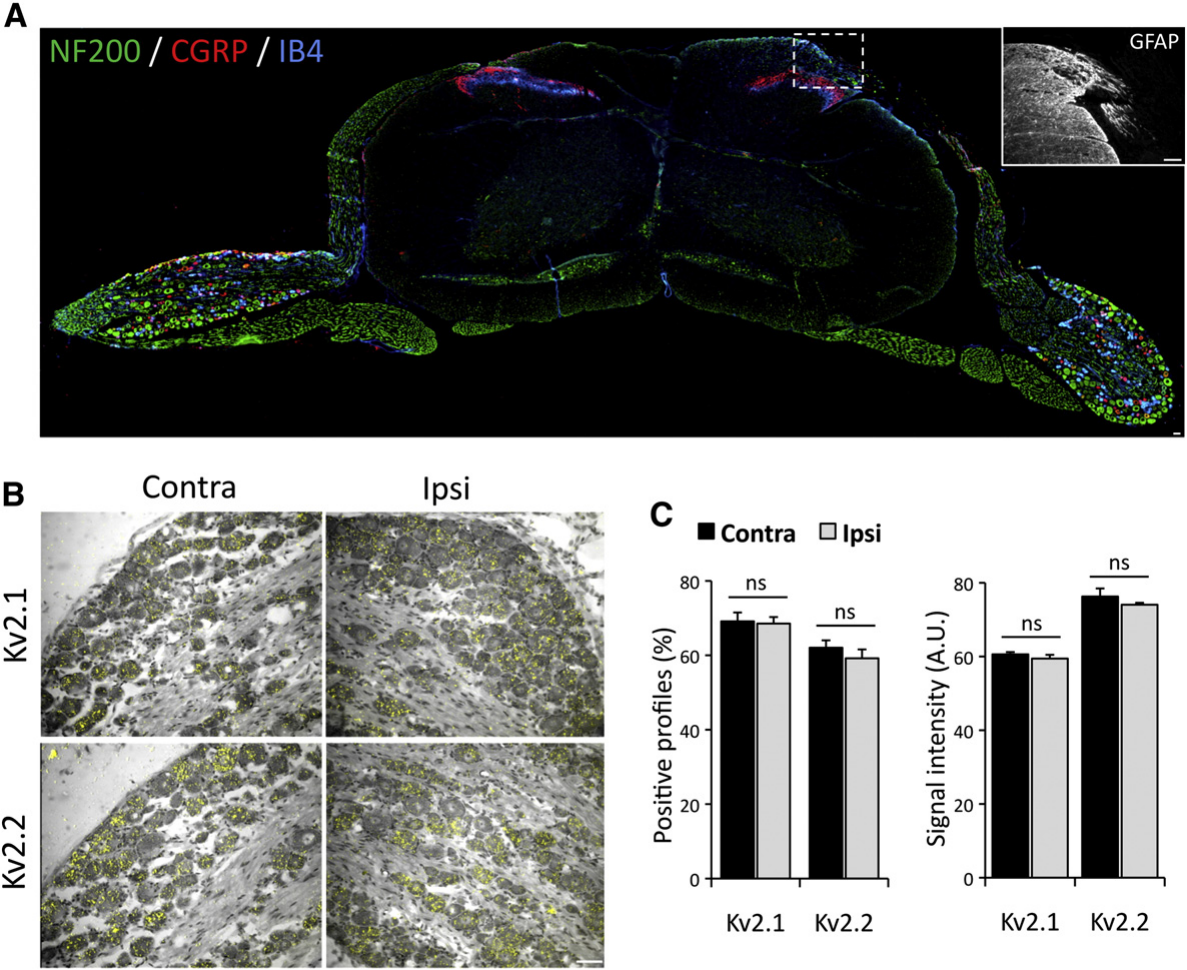
Kv subunits are not regulated by dorsal rhizotomy. (A) A transverse section of the spinal cord with DRGs attached, illustrating the dorsal rhizotomy of the right central processes, stained for NF200, CGRP and IB4. Inset, GFAP staining illustrating astrocyte activation at the dorsal root entry zone of the injured side. (B) Bright field and polarized images of DRG sections from the contralateral (left) or ipsilateral (right) side of rhizotomized animals (7 days), subjected to ISH for Kv2.1 (top) or Kv2.2 (bottom) mRNA. (C) Percentage of neurons positive for Kv2 mRNA expression and quantification of signal intensity with or without dorsal rhizotomy (mean ± SEM, n = 3 animals/group, paired *t*-test for ipsilateral *vs* contralateral sides for each subunit, *p < 0.005). Scale bars = 50 μm.

Given the dysregulation we observed after peripheral nerve injury, we asked whether injury of the central processes could inflict similar phenotypic changes. To achieve this, the dorsal rhizotomy model was used to compare Kv2 expression levels in DRG neurons of the injured (ipsi, right side in Fig. 4A) and uninjured (contra) sides, at 7 days after injury. Immunostaining for glial fibrillary acidic protein (GFAP) confirmed astrocyte activation at the ipsilateral dorsal root entry zone, indicating successful central axotomy (inset). Counts of Kv2 mRNA-containing neurons on the rhizotomized side were not significantly different compared to uninjured side (Kv2.1, 99.1 ± 2.5%; Kv2.2, 95.4 ± 3.8% of contra side; n = 3, paired *t*-test) (Fig. 4B & C). Quantification of silver grain intensity in those neurons also revealed no difference compared to control (Kv2.1, 98.1 ± 1.7%; Kv2.2, 97.1 ± 0.7% of contra; p > 0.05, n = 3, paired *t*-test) (Fig. 4C). In summary, Kv2 mRNA expression in the DRG is significantly reduced by peripheral axotomy but remains unaffected by rhizotomy.

### Kv2 dysregulation promotes DRG neuron hyperexcitability

**Fig. 5:**
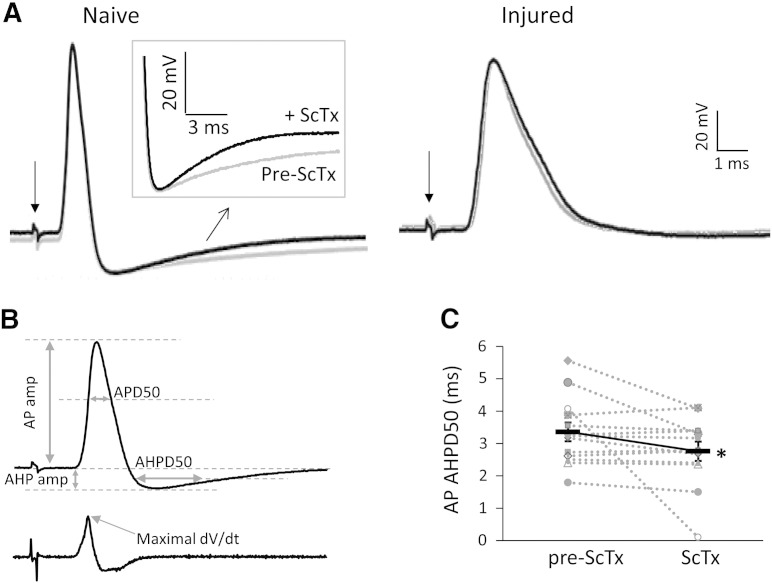
Effects of ScTx application on DRG neuron excitability. (A) Recordings from naïve and SNT-injured neurons showing evoked AP after dorsal root stimulation (indicated by the arrow) in the absence (grey) or presence (black) of ScTx. The inset shows the AHP on a larger scale. In naïve the AHP duration is shortened upon ScTx application, however in injured AHP is unaffected by ScTx application (B) Top, markers denoting the AP parameters calculated. Bottom, derivative of differentiated AP from top; arrow indicates the maximal rising rate. (C) Paired data demonstrating that treatment of naïve neurons with ScTx decreases the duration of after-hyperpolarization. The continuous black line connects mean ± SEM (n = 13, *p < 0.05, paired *t*-test).

To further investigate the involvement of Kv2 dysregulation in the electrophysiological properties of myelinated DRG neurons, we setup *ex vivo* intracellular DRG recordings (Fig. 5A & Table 5). The conduction velocity range for neurons analysed was 4.83–26.98 m/s, indicating that they were medium to large sized neurons (McCarthy and Lawson, 1990). We initially examined biophysical parameters of the APs evoked by dorsal root stimulation, including AP amplitude (AP amp), AP half width (APD50), AP after-hyperpolarisation amplitude (AHP amp) and half width (AHPD50) (described in Fig. 5B). In injured neurons, APD50 was dramatically increased compared to naïve (0.73 ± 0.11 ms *vs* 1.27 ± 0.12 ms, n = 13 per group; p < 0.001, Mann–Whitney U test), suggesting a much slower repolarisation. The amplitude of AHP was also significantly reduced in injured neurons (− 8.85 ± 1.01 mV *vs* − 13.25 ± 1.24 mV, n = 13 per group, p < 0.05, Mann–Whitney U test). These changes are consistent with previous reports of reductions in various Kv conductances in injured neurons (Chien et al., 2007, Kim et al., 2002, Park et al., 2003, Rasband et al., 2001). We also observed a decreased maximal rising rate in injured neurons (362.22 ± 38.93 V/s *vs* 242.31 ± 27.24 V/s, n = 13 per group, Mann–Whitney U test), in line with previously documented alterations in the expression, trafficking and kinetic properties of sodium channels (Devor, 2006). These changes were not associated with any change in input resistance or resting membrane potential. Other parameters like AP amp and AHPD50 were not altered by injury (Table 5).

**Table 5:** Comparison of electrophysiological parameters before and upon ScTx application in naïve and SNT-injured DRG neurons.

Naive	RMP	IR	AP amplitude	APD50	Max rising rate	AHP amplitude	AHPD50
Pre-ScTx	− 65.68 ± 1.64	14.56 ± 1.65	90.97 ± 3.55	0.73 ± 0.11	362.22 ± 38.93	− 13.25 ± 1.24	3.36 ± 0.29
ScTx	− 65.04 ± 2.55	16.61 ± 3.28	85.73 ± 3.16	0.77 ± 0.11	313.27 ± 30.52	− 13.27 ± 1.46	2.76 ± 0.30⁎



SNT	RMP	IR	AP amplitude	APD50	Max rising rate	AHP amplitude	AHPD50
Pre-ScTx	− 62.67 ± 1.01	21.06 ± 2.52	83.94 ± 3.36	1.27 ± 0.12###	242.31 ± 27.24#	− 8.85 ± 1.01#	4.02 ± 0.20
ScTx	− 60.89 ± 1.23	20.61 ± 3.31	80.92 ± 2.63	1.45 ± 0.20	218.09 ± 20.37	− 8.44 ± 1.14	3.86 ± 0.21

RMP: resting membrane potential, in mV; IR: input resistance, in MΩ; AP amplitude: in mV; APD50: AP half width, in ms; Maximal rising rate: in V/s; AHP: after-hyperpolarization; AHP amplitude: in mV; AHPD50: AHP half width, in ms. N = 13 for all data. Statistics for paired data in naïve or SNT groups were performed using paired *t*-test. All comparisons between naïve and SNT before ScTx application were done by using Mann–Whitney *U* test.

⁎ p < 0.05.

# p < 0.05.

### p < 0.001.

To further investigate the involvement of Kv2 dysfunction in DRG neuron excitability, we utilised the Kv2 channel gating modifier ScTx, which shifts Kv2.1 and Kv2.2 channel activation towards more depolarized potentials (Bocksteins et al., 2009). Application of ScTx to naïve neurons did not cause any changes in AP amp, APD50, maximal rising rate or AHP amp, in accordance with the relatively slow activation kinetics of Kv2 conductance (Johnston et al., 2010). However, ScTx reduced AHPD50 by 18% (3.36 ± 0.29 ms *vs* 2.76 ± 0.30 ms, n = 13, p < 0.05, paired *t*-test; Fig. 5A & C), consistent with a role of Kv2 in the repolarization and hyperpolarisation phases. In contrast, recordings from SNT injured neurons (Fig. 5A, right) showed that AHPD50 and all other examined parameters remained unaffected by ScTx treatment (pre-ScTx, 4.02 ± 0.20 *vs* ScTx, 3.86 ± 0.21 ms; p > 0.05, paired *t*-test). This result suggests that a substantial reduction of Kv2 conductance is already established in injured neurons, in accordance with the Kv2 downregulation we documented following axotomy. Finally, the AHPD50 following injury was not significantly different compared to naïve (n = 13, p > 0.05, Mann–Whitney *U* test). Given the documented Kv2 downregulation by injury and the shortening of AHPD50 by Kv2 inhibition, a reduced AHPD50 might be expected. However, the neuropathology associated with nerve lesions is characterized by parallel dysregulation of multiple ion channels. Thus, other injury-induced changes in conductances involved in after-hyperpolarization, like those of Kv1, Kv3 (Johnston et al., 2010), Ca^+ 2^-activated potassium channels (Scholz et al., 1998), and hyperpolarization-activated cyclic nucleotide-gated (HCN) channels, may mask the Kv2 effect on AHPD50.

**Fig. 6:**
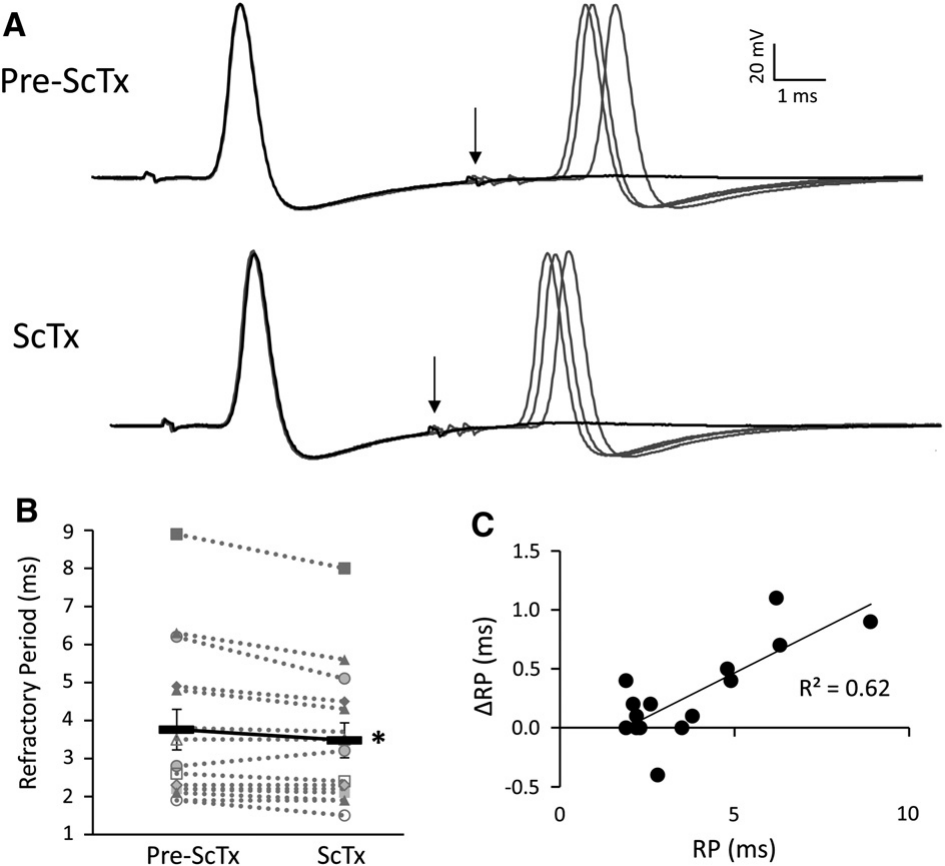
ScTx treatment shortens the refractory period of DRG neurons. (A) Representative traces illustrating that refractory period is shortened upon ScTx application. The refractory period was defined as the maximal inter-pulse interval at which the second stimulus fails to elicit an AP at a strength of 2 × threshold current. In naïve neurons (top), black trace shows that the second AP fails when interval is 6.2 ms (arrow). Following ScTx application however (bottom), the second AP fails at the interval of 5.1 ms (arrow). Inter-pulse intervals successfully eliciting APs are shown in grey and correspond to 6.3, 6.5, and 7.0 ms (pre-ScTx) and 5.2, 5.5, and 5.8 ms (ScTx). (B) Paired data showing refractory period in naive uninjured neurons is significantly shortened by ScTx (continuous black line indicate mean ± SEM, n = 15, *p < 0.05, paired *t*-test). (C) Correlation between refractory period before ScTx application and the change upon ScTx application in naive neurons (r = 0.79, p < 0.001, Pearson's correlation test).

The observed reduction in after-hyperpolarization duration by ScTx in naïve neurons is postulated to shorten inter-spike intervals during repetitive discharge. To further address this hypothesis, we measured the AP refractory period (RP) in naïve neurons, before and upon ScTx application (Fig. 6A). Indeed, ScTx treatment led to a significant reduction in RP, from 3.76 ± 0.54 ms to 3.48 ± 0.48 ms (n = 15, p < 0.05, paired-*t*-test; Fig. 6B). This reduction was more evident in neurons with longer baseline RP, illustrated by the correlation between baseline RP and relative change upon ScTx application (Fig. 6C; r = 0.79, p < 0.001, Pearson's correlation test). This finding demonstrates that in DRG neurons RP duration is associated with the amount of Kv2 current. Thus, the more Kv2 conductance present in a neuron, the wider the AHPD50 and longer the refractory period, and *vice versa*.

**Fig. 7:**
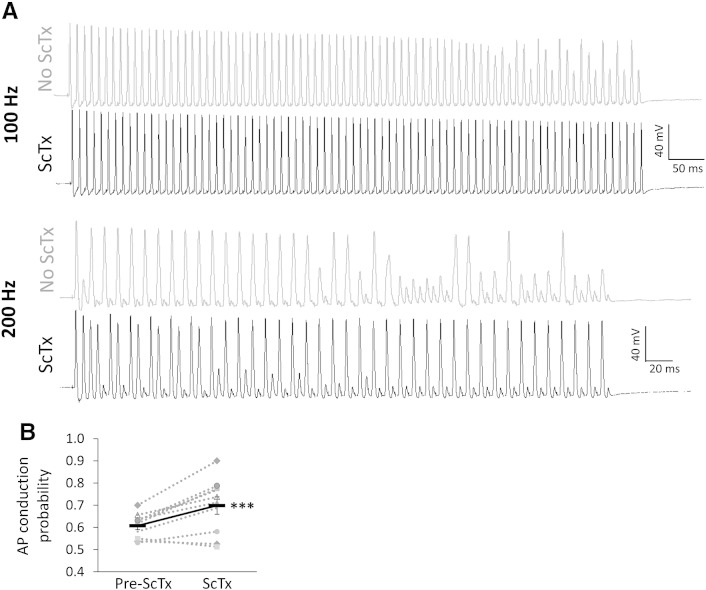
ScTx application enhances AP conduction during prolonged stimulation. (A) Naive DRG neuron responses to a train of 80 stimuli delivered at either 100 Hz (top) or 200 Hz (bottom), before or after ScTx application. At both frequencies, repetitive stimulation eventually causes AP conduction failure (100 Hz, failed after 50–60 stimuli; 200 Hz, failed regularly every other stimulus but more so after 40 stimuli). In the presence of ScTx however the fidelity of the response is improved and the neuron can more efficiently follow the stimulation train at both frequencies. (B) Quantification of AP conduction probability before and after ScTx treatment. The continuous black line connects mean ± SEM (n = 10, ***p < 0.005, paired *t*-test).

Individual APs represent the basic unit of neuronal signalling, whereas sensory communication and chronic pain in particular depend on sustained firing. We therefore investigated the direct effect of Kv2 inhibition on the ability of myelinated neurons to faithfully conduct APs following repetitive stimulation (Fig. 7). In normal conditions, failure of AP conduction to the soma was observed after approximately 50–60 stimuli at 100 Hz. Increasing the stimulation rate to 200 Hz caused AP failure initially at every other stimulus and even more frequently after the first 40 stimuli (Fig. 7). Upon ScTx application however, the fidelity of the response was substantially improved at both frequencies and neurons followed the stimulation train much more efficiently. Thus, quantification of the AP conduction probability showed a significant increase following ScTx treatment (0.70 ± 0.04 *vs* 0.61 ± 0.02; n = 10, paired *t*-test, p < 0.001). This result is in line with the notion that Kv2 dysfunction in chronic pain facilitates the high firing rates of injured primary afferents, triggered either spontaneously or following stimulation. Taken together, our data suggest that injury-induced Kv2 downregulation confers electrophysiological changes that underlie important aspects of the hyperexcitable phenotype encountered in neuropathic pain states.

## Discussion

Several studies have provided evidence for differential expression of Kv subunits in diverse classes of sensory neurons (Gold et al., 1996, Rasband et al., 2001). The distinct distribution, combined with subunit tetramerization and extensive post-translational modifications equips sensory neurons with a sophisticated machinery to differentially encode and respond to varying intensities of stimuli. In support of such a functional diversity, up to six pharmacologically and kinetically distinct K^+^ currents have been recorded in DRG (Gold et al., 1996). Some of these currents are specifically detected in neurons of defined sizes, while other studies suggest that even within a restricted functional group, such as large cutaneous afferent neurons, there is considerable variation in the biophysical characteristics of recorded K^+^ currents (Everill et al., 1998). A distinction between neurons of different sizes and specific K^+^ currents has also been reported in trigeminal ganglia (Catacuzzeno et al., 2008). However, electrophysiological assessment of K^+^ currents can often be inconclusive for the precise identification of contributing channels, due to the overlapping pharmacology (Johnston et al., 2010), modifications introduced by phosphorylation (Misonou et al., 2005) and interactions with auxiliary partners (Kerschensteiner and Stocker, 1999, Pongs and Schwarz, 2010, Vacher and Trimmer, 2011). Therefore, a supplementary classification based on expression of potassium channel subunits can further elucidate the underlying associations.

This study provides the first comprehensive characterisation of Kv2 subunit expression in DRG neurons. Kv2.1 and Kv2.2 were detected in cells of all sizes, and were particularly abundant in medium-large NF200 neurons which give rise to A-fibres. These include the Aδ nociceptors signalling mechanical and heat pain and the Aβ fibres, which are predominantly low-threshold mechanoreceptors. Although Aβ fibres do not contribute to painful sensations under physiological conditions, they become spontaneously active after neuropathic lesions (Calvo and Bennett, 2012, Kajander and Bennett, 1992, Liu et al., 2000, Michaelis et al., 2000). The spontaneous activity in A fibres can trigger central sensitization in the spinal cord, which amplifies the input and contributes to neuropathic pain sensations (Michael et al., 1999, Molander et al., 1994, Noguchi et al., 1995). Kv2 subunits were also expressed in approximately half of small unmyelinated neurons. These were identified as peptidergic and non-peptidergic nociceptors, which encode multiple pain modalities and have an established role in chronic pain syndromes. A corresponding delayed rectifier current modulated by ScTx has been detected in small nociceptors and this represented the majority of sustained Kv conductance *in vitro* (Bocksteins et al., 2009). In summary, the expression pattern we detected supports a physiological role for Kv2 subunits in both small and medium-large sensory neurons.

A substantial body of work has established an association between reductions in potassium currents and enhanced excitability of primary sensory neurons (Abdulla and Smith, 2001, Everill and Kocsis, 1999, Tan et al., 2006). Thus previous studies have related aspects of the altered phenotype to downregulation of Kv1 (Park et al., 2003, Yang et al., 2004, Zhao et al., 2013), Kv4 (Cao et al., 2010, Chien et al., 2007) or Kv7 (Rose et al., 2011) or Kv9 (Tsantoulas et al., 2012) subunits in sensory neuron subsets. Our study complements these by relating diminished Kv2 mRNA expression and function to specific electrophysiological changes following traumatic nerve injury. Both Kv2.1 and Kv2.2 subunits showed a rapid and uniform transcriptional downregulation in all cell types commencing within 24 h post-injury, while the bulk of expressional changes were established by day 3 and were long-lasting, coinciding with the onset of hyperexcitability and pathophysiological pain in this model (Kajander et al., 1992, Liu et al., 2000). A limitation of this study is that only mRNA levels were assessed. Although transcriptional downregulation typically (but not always) results in concomitant reductions in the encoded protein, the magnitude of the effect can vary considerably (Vogel and Marcotte, 2012). More importantly, the current analysis does not allow determination of whether changes in Kv2 protein precede the establishment of pain phenotypes. Supplementary investigations using specific antisera to Kv2 subunits should clarify these questions. Nevertheless, a diminished Kv2 function once pain is established is in agreement with the finding that ScTx application 7 days following injury did not affect the biophysical properties of axotomized neurons, as determined *via* intracellular recordings.

Consistent with the putative role of Kv2 downregulation in neuropathic pain, we found no change in Kv2 mRNA 7 days after dorsal root rhizotomy, a procedure that does not produce hyperexcitability (Sheen and Chung, 1993, Yoon et al., 1996) or pain behaviours in rodents and humans (Loeser, 1972, Sukhotinsky et al., 2004). Although it is possible that rhizotomy led to more transient alterations that had already recovered by that time, previous studies suggest that hallmark changes in this model, such as glial marker induction, are established as early as day 2 and persist for at least 14 days (Chew et al., 2011). In line with this, GFAP immunoreactivity at 7 days revealed astrocyte infiltration, reflecting the formation of a non-permissive glial scar at the injury site.

Kv2 channels are activated slowly after large membrane depolarisations and therefore do not generally affect spike thresholds. However, during AP firing Kv2 opening contributes to membrane repolarisation and hyperpolarization back to resting potential. Furthermore, the characteristic slow kinetics of activation and inactivation mean that the role of Kv2 becomes more pronounced during sustained input, due to the cumulative recruitment of activated channels. Indeed, Kv2.1 has a key role in controlling somatodendritic excitability of hippocampal neurons during high frequency input (Du et al., 2000), while Kv2.2 conductance regulates excitability of medial nucleus of the trapezoid body neurons during sustained firing by hyperpolarising inter-spike potential and thus allowing sodium channels to recover more quickly from inactivation (Johnston et al., 2008). Interestingly, in our experiments Kv2 inhibition in sensory neurons did not affect the amplitude of after-hyperpolarisation but reduced its duration, suggesting a slightly different mechanism. Importantly, this reduction in the after-hyperpolarisation phase was also associated with a decrease in the AP refractory period. We reasoned this shortening of spike intervals could accommodate higher firing rates. Indeed, when we challenged the neurons with a train of stimuli we discovered that Kv2 inhibition improved the fidelity of AP conduction in the DRG soma during sustained high frequency stimulation. In hippocampal and cortical neurons, the dominant effect that Kv2 channels exert on conduction is assisted by their specific localisation in the axon initial segment, where they act as a bottleneck low-pass filter to control AP output (Hwang et al., 1993, Sarmiere et al., 2008). Whether such particular axonal targeting also exists in primary sensory neurons is currently unknown, but is a tempting possibility given the influence of branching points on DRG impulse conduction (Stoney, 1990). The lack of any ScTx effect on the repolarisation and after-hyperpolarisation phases in injured cells suggests that conduction probability would remain unaffected by ScTx treatment, although we did not directly test this hypothesis. Future validation of this would further support a causal link between Kv2 dysfunction and conduction changes in axotomised neurons.

Our study is the first to demonstrate that blocking Kv2 channels in A-fibres enhances conduction fidelity. Although we only assessed medium-large neurons, the finding that Kv2 subunits are also substantially downregulated in unmyelinated neurons creates the possibility that a similar mechanism may affect C-fibre excitability. The downstream effects of Kv2 dysfunction could be even more pronounced in C-fibres, since these afferents are particularly reliant on conduction of impulses at high-frequency during pain signalling. Such enhanced C-fibre activity during sustained stimulation could feed the spinal cord with a barrage of impulses that drives central sensitisation, and thus mediates exaggerated pain sensations (Raja et al., 1988, Wu et al., 2001). Intriguingly, changes in C- and A-fibre following frequency due to reduced conduction failure have also been described in non-traumatic models of pain, such as osteoarthritis and diabetic neuropathy (Sun et al., 2012, Wu and Henry, 2013). Taken together, these results put forward the hypothesis that under physiological conditions Kv2 channels act as an essential excitability brake in sensory neurons. Diminished Kv2 function due to axotomy or pharmacological blockade contributes to neuronal hyperexcitability by promoting repetitive firing driven by sustained input. Besides direct stimulation, another likely source of such heightened input is the spontaneous activity that typically develops in neuropathic pain states (Kajander and Bennett, 1992, Liu et al., 2000). Interestingly, Kv2.2 dysfunction in cortical neurons also induces pain hypersensitivity, indicating that normal Kv2 activity may be instrumental at higher levels of the nervous system as well (Thibault et al., 2012).

We have previously reported that diminished function of Kv9.1, a modulatory subunit of Kv2 that is exclusively expressed in myelinated DRG neurons, leads to pain behaviours linked to augmented spontaneous and evoked firing and persistent after-discharge (Tsantoulas et al., 2012). Interestingly, *in vivo* inhibition of Kv9.1 also reduces the after-hyperpolarisation duration in the same fashion that Kv2 inhibition by ScTx did (Tsantoulas et al., 2012). Combined, these two studies strongly \suggest that the downstream effect of Kv9.1 silencing is reduced Kv2 conductance, which in turn causes profound excitability changes during sustained input and pain phenotypes. Since Kv9.1 has been shown to associate with Kv2 subunits in heterologous systems (Salinas et al., 1997b), one interpretation for these effects is the elimination of a Kv9.1/Kv2.x heterotetramer; however more work is needed to decipher the exact Kv2 heterotetramer composition and properties in DRG neurons.

The molecular injury-induced trigger that controls Kv2 expression remains elusive. The divergent Kv2 regulation in peripheral *versus* central axotomy may be indicative of the involvement of a peripheral target-derived trophic factor. Although not systematically tested yet, there is indeed some data suggesting that Kv regulation by neurotrophins is physiologically relevant (Cao et al., 2010, Everill and Kocsis, 2000, Park et al., 2003, Sharma et al., 1993, Zhu et al., 2012). Interestingly, it was recently found that injury-induced Kv1.2 down-regulation and associated pain behaviours can be reversed by targeting an endogenous non-coding RNA which modulates Kv1.2 expression in DRG (Zhao et al., 2013). Given the degree of conservation amongst Kv channels it is plausible that similar mechanisms also govern Kv2 expression. Additionally, Kv2.1 conductance is regulated by AMIGO, an auxiliary subunit that co-localises with Kv2.1 in the brain (Peltola et al., 2011). Whether AMIGO or other yet unidentified proteins exert similar roles in the peripheral nervous system remains to be determined.

Our results suggest that nerve injury does not completely ablate Kv2 expression, which has implications for treatment. Developing specific openers to target residual Kv2 expression could compensate the loss-of-function, dampen neuronal activity and thus provide pain relief following nerve lesions, similarly to Kv7 openers (Blackburn-Munro and Jensen, 2003, Dost et al., 2004, Mishra et al., 2012, Roza and Lopez-Garcia, 2008). The same endpoint could be accomplished *via* activation of the PKC, CDK5, Src and AMP-activated protein kinases, since Kv2 phosphorylation can facilitate Kv2 currents and reduce excitability (Cerda and Trimmer, 2011, Ikematsu et al., 2011, Park et al., 2006, Song et al., 2012). Lastly, instigation of a recently identified nitric oxide signalling cascade can also robustly increase Kv2 currents in CNS neurons (Steinert et al., 2011).

In conclusion, Kv2 activity appears to be a key component that helps fine-tune neuronal excitability. Pharmacological modulation of this activity may create novel therapeutic opportunities for neurological disorders and chronic pain management in particular.
